# Effect of Baihu and Guizhi decoction in acute gouty arthritis: study protocol for a randomized controlled trial

**DOI:** 10.1186/s13063-022-06194-z

**Published:** 2022-04-15

**Authors:** Yikun He, Chaoran Dai, Jiaying Shen, Qianwen Chen, Jiandong Gao, Xin Pan, Jing Gan

**Affiliations:** 1 Department of Rheumatism, Shuguang Hospital Affiliated to Shanghai University of Traditional Chinese Medicine, Shanghai, 201203 China; 2 Shanghai University of Traditional Chinese Medicine, Shanghai, 201203 China; 3 Department of Ultrasonography, Shuguang Hospital Affiliated to Shanghai University of Traditional Chinese Medicine, Shanghai, 201203 China; 4 Department of Nephrology, Shuguang Hospital Affiliated to Shanghai University of Traditional Chinese Medicine, TCM Institute of Kidney Disease, Shanghai University of Traditional Chinese Medicine, Key Laboratory of Liver and Kidney Diseases (Shanghai University of Traditional Chinese Medicine), Ministry of Education, Shanghai Key Laboratory of Traditional Chinese Clinical Medicine (14DZ2273200), Shanghai, 201203 China

**Keywords:** Baihu and Guizhi decoction, Acute gouty arthritis, Randomized controlled trial

## Abstract

**Background:**

The prevalence rates of gout worldwide have increased annually. Acute gouty arthritis (AGA) accounts for a large proportion of gout patients and causes severe physical and mental pain in patients. Controlling the occurrence and development of gout inflammation is the first step in the treatment of gout. The main treatment drugs in gout are non-steroid anti-inflammatory drugs (NSAIDs), colchicine, and glucocorticoids, but these treatments have many adverse reactions which limit their clinical application. Baihu and Guizhi decoction (BHGZ) is one of the classic prescriptions in the Synopsis of the Golden Chamber and is a good prescription for AGA. Previous clinical studies have shown that BHGZ confers a strong benefit for treating AGA. However, the literature shows a lack of high-quality RCT research on BHGZ with respect to AGA. Therefore, in this study, we use a randomized, double-blind, controlled study with a placebo to evaluate the clinical efficacy and safety of BHGZ on the AGA of moist heat arthralgia spasm syndrome.

**Methods:**

This study is a randomized, double-blind, controlled clinical trial. A total of 102 adult participants with AGA of moist heat arthralgia spasm syndrome will be enrolled, with balanced treatment allocation (1:1). The experimental intervention will be BHGZ plus the low-dose colchicine, and the control intervention will be placebo plus the low-dose colchicine for 10 days. To study the clinical efficacy (including VAS score; joint tenderness, joint swelling, joint movement disorder; TCM evidence efficacy score) and the changes of inflammatory indexes. At the same time, the improvement of joint inflammation in patients with AGA will be observed from musculoskeletal ultrasound imaging, and the safety evaluation will be carried out.

**Discussion:**

This study will be the first placebo-controlled RCT to assess whether BHGZ plus low-dose colchicine have beneficial effects on changing reducing inflammation of joints for patients with AGA of moist heat arthralgia spasm syndrome. The results of this trial will help to provide evidence-based recommendations for clinicians.

**Trial registration:**

Chinese Clinical Trials Register ChiCTR1900024974. Registered on 5 August 2019

**Supplementary Information:**

The online version contains supplementary material available at 10.1186/s13063-022-06194-z.

## Background

Gout is a metabolic inflammatory disease caused by the deposition of monosodium urate (MSU) in the cartilage, synovial sac, tendon, soft tissue, and/or kidney. Gout is directly related to hyperuricemia, potentially attributable to changes in lifestyle and diet. The prevalence rates of gout worldwide have increased annually [1]. In the USA, the morbidity of gout is 3.76%, and in Britain, it is 2.49% [2]. The prevalence of gout in China is 1–3% and shows an increasing trend, particularly among younger people, with lower incidence in inland than in coastal areas and a higher prevalence in males than in females [3].

The development of gout includes four stages: asymptomatic hyperuricemia, acute attack, remission, and chronic gouty arthritis (gout stones). An acute attack of gout is closely related to the rapid fluctuation of serum uric acid. In acute attacks, the rapid development of severe pain, swelling, and tenderness can reach its maximum within 6–12 h of the immune response being triggered by urate. Thus, the early treatment of acute gouty arthritis (AGA) not only quickly improves the clinical symptoms but also reduces the inflammation and joint tissue damage mediated by the immune response. The aim of AGA treatment is attenuated inflammation and analgesic relief, and the main treatment drugs are non-steroid anti-inflammatory drugs (NSAIDs), colchicine, and glucocorticoids. The American College of Rheumatology (ACR) criteria and European League Against Rheumatism (EULAR) guidelines of 2015 also recommended the use of these three types of drugs [4]. However, these drugs are associated with many adverse reactions, such as colchicine-induced diarrhea and liver and kidney toxicity; NSAID-induced gastric mucosa damage and liver and kidney injury; and glucocorticoid-induced metabolic disorders and osteoporosis. These adverse reactions greatly limit their clinical application, particularly for patients with liver and renal insufficiency and acute gout. Therefore, it is necessary to explore additional rapidly effective and less adverse drugs in the treatment of AGA.

Chinese herbal medicine has been used in China for thousands of years. Syndrome differentiation and treatment are the main characteristics and therapeutic guidelines for traditional Chinese medicine (TCM). According to the diagnostic criteria of TCM, the analysis of clinical data is obtained from the four main diagnostic methods—observation, listening, interrogation, and pulse-taking. After years of clinical practice treating AGA, most traditional Chinese medicine scholars believe that moist heat arthralgia spasm syndrome is the common TCM syndrome of AGA [5]. The clinical manifestations are as follows: swollen small joints of the lower limbs, painful and heated acute attacks relieved by coolness and prevention of added pressure, fever, thirst, sweating, yellow urine, red tongue with yellowish or greasy fur, and rapid pulse. Baihu jia Guizhi decoction (BHGZ) from *the Synopsis of the Golden Chamber* can clear away heat, remove dampness, dredge collaterals, and relieve pain. It is a classic prescription for the treatment for the moist heat arthralgia spasm syndrome of gout in T*raditional Chinese Medicine Internal medicine* published by China Press of Traditional Chinese Medicine [6]. Previous clinical studies have shown that BHGZ confers a strong benefit for treating AGA [7, 8]. However, the literature shows a lack of high-quality RCT research on BHGZ with respect to AGA. Therefore, in this study, we use a randomized, double-blind, controlled study with a placebo to evaluate the clinical efficacy and safety of BHGZ on the AGA patients of moist heat arthralgia spasm syndrome. We hypothesized that BHGZ can effectively reduce the clinical symptoms and signs, improve quality of life, shorten the treatment time, quickly relieve joint inflammation, and demonstrate a good safety profile for use on AGA patients of moist heat arthralgia spasm syndrome.

## Methods

**Fig. 1:**
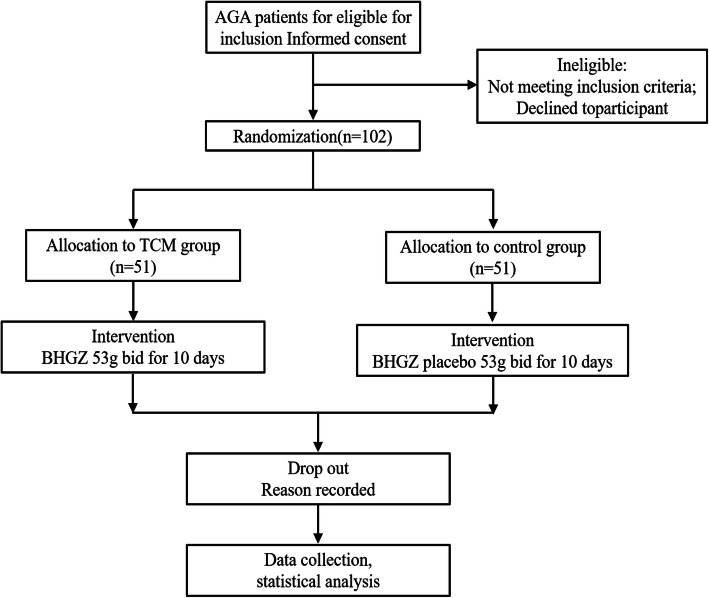
Study flow chart

This is a randomized, double-blind, controlled exploratory study with a 10-day follow-up. Each participant will be asked for written informed consent (Fig. 1).

### Sample size calculation

To determine the sample size, we used the findings from previous studies. The rate of colchicine effectiveness in the treatment of AGA was 67% [9] and that of BHGZ was 91% [8]. When *α* = 0.05 and the test efficacy 1−*β* = 0.80, the sample size needs to be *n* = 81, with 41 in each treatment group. Considering a 20% dropout rate, the planned sample size for randomization is increased to 102. The formula for calculation is as follows:

*n* = (*U*_*α*_ + *U*_*β*_)^2^(1 + 1/*k*)*P*(1−*P*)/(*P*_1_−*P*_2_)^2^

This study will recruit participants from Shuguang Hospital Affiliated with the Shanghai University of TCM. An advertisement encouraging clinical trial enrollment will be posted on the webpage. We plan to recruit patients from participating hospitals via poster, networking, or WeChat. The posters will be placed on bulletin boards or other assigned places in the hospitals. At least one staff member (a postgraduate or doctor) will specialize in patient recruitment. Their contact information and clinic for screening visit will be detailed described in the recruitment advertisement.

### Diagnostic criteria for AGA

The diagnosis of acute gout arthritis is made according to the 2015 ACR criteria and EULAR Classification Criteria for AGA [4].

### Diagnostic criteria for TCM syndrome differentiation

The diagnosis of “moist heat arthralgia spasm syndrome” is based on the following diagnosis and treatment routines of TCM syndromes in *Traditional Chinese Medicine Internal medicine* published by China Press of Traditional Chinese Medicine [6]:

- Swollen small joints of the lower limbs and painful acute attack and heat which are relieved by application of coolness and prevention of added pressure, accompanied by fever, thirst, upset, and yellow urine
- Tongue and pulse: red and rolling tongue with yellow or greasy fur and rapid pulse

### Inclusion criteria

1. Adult subjects with a diagnosis of AGA as stated above and with a TCM diagnosis of moist heat arthralgia spasm syndrome
2. Males and females, aged 18–65 years, with the ability to act independently
3. Acute onset within 48 h
4. No other similar treatment within 14 days before the trial
5. Signed informed consent form

### Exclusion criteria

1. Pregnant or lactating women
2. Patients with stage IV–V chronic kidney disease (eGFR< 30 ml/min/1.73 m^2^)
3. Allergies to the tested drugs
4. Abnormal liver function: an ALT level that is higher than twice the normal level
5. Severe acute/chronic organic or mental diseases
6. Severe deformity or inability to be in the labor force because of late-stage arthritis
7. Poor compliance and/or inability to complete the clinical observation

### Rejection criteria

1. Failure to meet the criteria but mistakenly admitted
2. Failure to take medicine according to instruction during the trial, affecting the efficacy results
3. Patients who take other traditional Chinese medicine drugs that are prohibited by the instructions of the trial, interfering with the correct evaluation of the efficacy and safety

### Randomized and blind review

The randomization is computer generated by an independent statistician using SPSS software (SPSS version 21.0; IBM, NY, USA). Participants will be randomly assigned (on a 1:1 ratio) to a BHGZ group and placebo group. If the patients agree to participate and sign the informed consent voluntarily after screening, then an independent research physician, who is not engaged in the recruitment for this study, the treatment provided, or the assessments, will assign a random sequential number to the participant. The packaging of BHGZ or placebo drug will be labeled with the random sequential number according to a random code. The independent statistician will examine and verify the packaging of the drugs and placebo by the pharmaceutical company during labeling. Each patient will be distributed a 3-day dose with the lowest number of drugs or placebo by a clinical pharmacist, who does not know the allocation groups of the participants.

### Blind review

The trial will use the double-blind, placebo-controlled method. BHGZ granules and placebo granules have the same size, shape, color, and packaging. An independent statistician will divide BHGZ and placebo drugs into two types (A and B) and then seal the information to the bottom of the packaging, according to the random results. This blind method will be applied to both participants and researchers (including the physicians, investigators, coinvestigators, and pharmacists), and the drugs were distributed according to the visiting sequence. Unblinding will be performed according to the standard operating procedure (SOP) of the Contract Research Organization (CRO) [10]. If adverse events occur during the test, then the principal investigator will be given an emergency envelope to use as a backup, and a secondary blind method will be used, with the principal investigator reporting this process to the independent safety monitoring board. When the trial is completed, the independent statistician will lock the data after checking to ensure that the data cannot be modified. Next, the independent statistician who preserved and sealed the information to the bottom of the packaging will start the first level of unveiling and immediately inform the statistician about the corresponding group numbers for each enrolled patient. The statistician will conduct statistical analysis on all the collected data. After the completion of the statistical analysis of the data, the blind information will be revealed again. Finally, the groups corresponding to A or B will be announced by the independent statistician.

### Intervention

- Basic therapy

Two groups will follow a basic Western medicine treatment plan as indicated by the suggested use of colchicine in the Chinese gout diagnosis and treatment guide issued by the rheumatology branch of the Chinese Medical Association in 2016 [11]: A colchicine tablet taken orally (Kunming Pharmaceutical Group Co., Ltd.; 1.0 mg/tablet; H53021534) as follows: 1.0 mg initially, then 0.5 mg 1 h later, then 0.5 to 1.0 mg per day. If the participant has taken a uric-acid-lowering drug before entering the group, the drug and drug dosage will remain unchanged.

- Test drug name and specification

BHGZ granules comprise gypsum (Shigao; 30 g), *Rhizoma Anemarrhenae* (Zhimu; 9 g), prepared *Radix Glycyrrhizae* (Zhi Gancao; 3 g), raw Chinese yam (Sheng Shanyao; 6 g) instead of rice (Jingmi), and cassia twig (Guizhi; 5 g). The placebo comprises starch, dextrin, and bitter agent, which contain one-tenth of the content of BHGZ. Its smell and taste are similar to those of BHGZ granules to overcome the bias formed by researchers, subjects, and staff participating in the evaluation of efficacy and safety. BHGZ granules and the placebo granules are both manufactured by Jiangyin TianJiang Pharmaceutical Co., Ltd., and are used for the preparation of traditional Chinese medicine formula granules.

The composition and efficacy of each herb in BHGZ granules are summarized in Table 1.

**Table 1:** Composition and action of BHGZ granules in Chinese herbal medicine

Ingredients	Granule dose	Efficacy (TCM)	Efficacy (pharmaceutical study)
Gypsum (Shigao)	30 g	Clearing away heat and purging fire, myogenic, sore healing, removing dampness	1. Analgesic 2. Anti-inflammatory
Rhizoma Anemarrhenae (Zhimu)	9 g	Clearing away heat and purging fire, nourishing the kidney, and moistening dryness	1. Analgesic 2. Anti-inflammatory 3. Anti-tumor
Raw Chinese yam (Sheng Shanyao)	6 g	Tonifying the spleen and stomach, promoting fluid and benefiting lung, tonifying kidney and astringent essence	1. Antioxidant 2. Anti-aging 3. Immune regulation 4. Anti-tumor 5. Hypoglycemic
Cassia twig (Guizhi)	5 g	Resolving the flesh, harmonizing Ying and Wei	1. Antipyretic 2. Analgesic 3. Anti-inflammatory 4. Antiallergic 5. Antiviral microorganisms
Radix Glycyrrhizae (Zhi Gancao)	3 g	Tonifying the spleen and stomach, harmonizing the properties of various medicines	1. Antidepressant 2. Immune regulation 3. Arrhythmia regulation 4. Anti-tumor 5. Anti-inflammatory

### Outcome measures and time points

The general information, symptoms, and signs of the subjects will be collected at baseline (the day of enrollment) and on the 3rd, 7th, and 10th day in the treatment, and the relevant examinations will be carried out at the baseline and on the 10th day of treatment.

The general information includes the subjects’ age, gender, nationality, occupation, ID number, contact information, medical history, allergy history, family history and breath, body temperature, pulse, blood pressure, tongue coating, and pulse. The symptoms and signs will be measured using a VAS test, classification of joint tenderness, joint swelling and extent of joint movement disorder, and TCM syndrome curative effect score. The relevant examinations include laboratory tests and musculoskeletal ultrasonographic examinations. The laboratory tests include measurements of xanthine oxidase (chemical colorimetry) and serum uric acid (SUA, with an automatic biochemical analyzer), albumin/creatinine ratio, erythrocyte sedimentation rate (ESR, by the automatic ESR method), C-reactive protein (CRP), and levels of tumor necrosis factor-α (TNF-α), interleukin-6 (IL-6), IL-1, IL-10 (by ELISAs), NLRP3, nuclear factor kappa-B (NF-κB), and caspase-1 (by RT-PCR). These indicators will be measured at the Shuguang Hospital Medical Testing Center.

Musculoskeletal ultrasound will be performed by two experienced doctors. To reduce bias, the ultrasound doctors will be blinded to the clinical examination and laboratory results and prohibited from inquiring about a patient’s clinical symptoms during the examination. High-frequency ultrasound will be used to judge the synovial lesions in both knees, ankles, and first metatarsophalangeal joint of both feet. The synovial thickness, blood flow signal, resistance of blood flow signal, and double track sign will be measured. Each image will be saved, and the grade and total score of the detected lesions will be recorded.

The measured items and time points of data collection are found in Table 2.

**Table 2:**
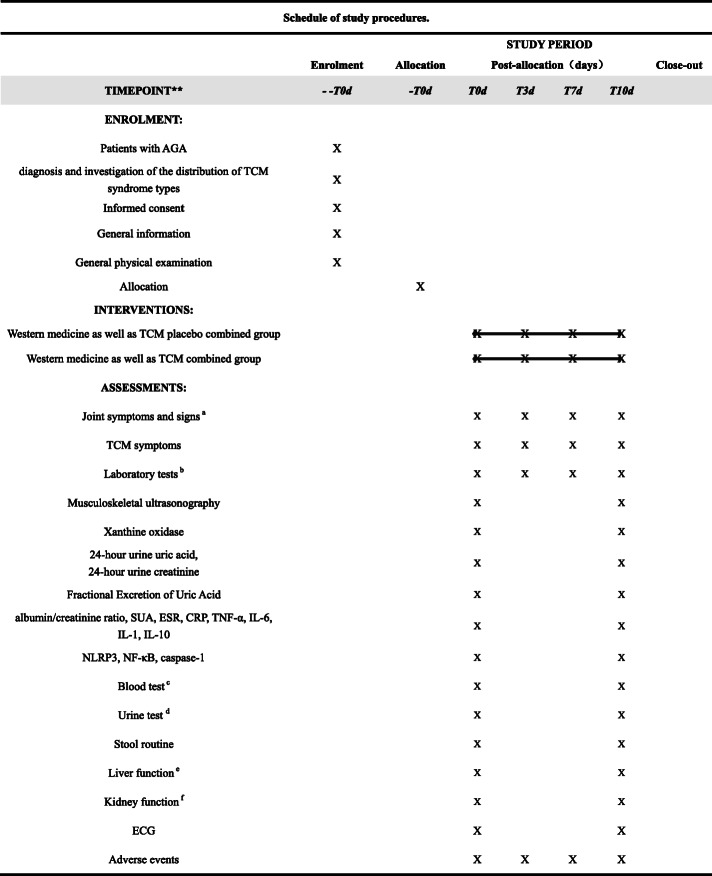
Schedule of study procedures

^a^Joint symptoms and signs include VAS test, joint tenderness, joint swelling, and joint movement disorder

^b^Laboratory tests include xanthine oxidase, 24-h urine uric acid, 24-h urine creatinine, uric acid excretion rate, fractional excretion of uric acid (FEua), albumin/creatinine ratio, SUA, ESR, CRP, TNF-α, IL-6, IL-1, IL-10, NLRP3, NF-κB, and caspase-1

^c^Blood test consists of white blood cell count (WBC), red blood cell count (RBC), hemoglobin (Hb), and platelets (PLT)

^d^Urine test consists of the WBC, RBC, and protein

^e^Liver function consists of alanine aminotransaminase (ALT), aspartate aminotransaminase (AST), gamma glutamyl (γ-GT), and alkaline phosphatase (AKP)

^f^Kidney function consists of serum creatinine (Scr) and blood urea nitrogen (BUN)

### Outcomes

The primary outcomes are the VAS test and changes in TCM symptoms. Secondary outcomes include the improvement of joint symptoms and signs (joint tenderness, joint swelling, and joint movement disorder), ESR, CRP, xanthine oxidase, 24-h urine uric acid, and 24-h urine creatinine levels; the uric acid excretion rate, FEua; ACR; and SUA, TNF-α, IL-6, IL-1, IL-10, NLRP3, NF-κB, caspase-1 levels, and evaluation of the joints via musculoskeletal ultrasound. Above these outcomes will be compared with those before treatment. Composite outcomes will include the incidence rates of all the possible safety events in the two groups. AEs will be monitored throughout the trial, and several biological indicators (blood, urine, and stool tests; routine liver and kidney function tests; and ECG tracings) will also be closely monitored.

### Measurement scale for TCM symptoms

To facilitate the evaluation of the results, we will use the measurement scale for TCM symptoms recommended by the Guidelines for Clinical Research of Chinese Medicine (New Drug) [12] to score the symptoms of the patients. The scale is based on 4 major symptoms and 7 minor symptoms. Each symptom is graded on 4 levels: as none, light, medium, and heavy with corresponding scores of 0, 1, 2, and 3, respectively. The total score is calculated by adding each score in the scale to calculate an efficacy indicator (EI) that is used to evaluate treatment efficacy:

EI = (Total symptom score at baseline − Total symptom score post-treatment)/Total symptom score before treatment × 100%.

The outcomes were categorized as full recovery: EI ≥ 90%, good recovery: 70% ≤ EI < 90%, modest recovery: 30% ≤ EI < 70%, and no recovery: EI < 30%.

### Evaluation of joint symptoms and signs

1. The visual analog scale (VAS) is presented on a 10-cm line with anchor statements on the left (no pain) and on the right (extreme pain).
  The patient is asked to mark their current pain level on the line. They may also be asked to mark their maximum, minimum, and average pain. The examiner scores the VAS by measuring the distance in either centimeters (0 to 10) or millimeters (0 to 100) from the “no pain” anchor point.

- 0 points: A distance of less than 1 cm indicates the current pain level
- 1 point: A distance between 1 and 4 cm indicates mild pain but with the ability to engage in daily activities
- 2 points: A distance between 4 and 7 cm indicates moderate pain with daily activities being affected
- 3 points: A distance between 7 and 10 cm indicates severe pain in daily life with restricted daily activities

The mitigation criteria for AGA: Response to BHGZ or placebo drug treatment will be determined from clinical symptoms and signs of subjects, as well as recorded pain scores of VAS. Responses are rated on a scale of no response, partial response, significant response, and complete resolution. Partial response is denoted by the documented clinical response by the clinician; the patient had “modest,” “some,” or “mild” improvement; or situations in which one joint is improved but another is not. The significant response is denoted by any combination of the following: functional improvement, such as the ability to walk or bear weight on the affected joint; movement or range of motion of the affected joint with minimal pain; improved swollen or tender joints on exam; or documented clinical response such as “dramatic” or “significant” improvement. If there is no documentation of the above, then a change in the average daily pain score by at least two points is considered a significant response; complete resolution is denoted that the patient’s flare is completely resolved [13].

(2) Evaluation of joint tenderness

- 0 points: No pain when under heavy pressure or performing passive activities
- 1 point: Mild pain: the patient claims tenderness with heavy pressure on the edge of the joint or with touch, but passive movement is not restricted
- 2 points: Moderate pain: The patient can feel pain with heavy pressure and expresses discomfort. Passive movement is sometimes restricted
- 3 points: Severe pain: The patient can feel pain with heavy pressure and tenderness and retreat. Passive mobility is severely restricted

(3) Evaluation of joint swelling

- 0 points: No joint swelling
- 1 point: The joints are slightly swollen, and the skin texture is lighter. The bone marks of the joint are still obvious
- 2 points: The joints are moderately swollen. Joint swelling is obvious. Most skin texture disappears, and most bone marks are not obvious
- 3 points: The joints are severely swollen, and the skin is tight with the loss of bone marks

(4) Evaluation of joint mobility

- 1 point: Activities are slightly restricted, but the patient can participate in normal activities
- 2 points: Obvious restricted activities. Normal activities are not permitted, but the patient can perform self-care
- 3 points: Activities are severely restricted, and the patient cannot perform self-care

(5) Evaluation criteria of the synovial thickness and blood flow signal by musculoskeletal ultrasound

The synovial thickness and blood flow signal are graded using the semi-quantitative method

a. The synovial thickness is divided into 4 grades [14]:

- Grade 1: no synovial hyperplasia with < 2-mm thickness
- Grade 2: mild synovial hyperplasia with 2- to 4-mm thickness
- Grade 3: moderate synovial hyperplasia with 5- to 9-mm thickness
- Grade 4: synovial hyperplasia with > 9-mm thickness

b. The blood flow signal levels are divided into 3 grades [15]:

- Grade 0: no signal, no blood flow
- Grade 1: mild, single-vessel signal or independent signal, ≤3 independent signals
- Grade 2: medium, fused vessels; > 3 independent signals or less than one-half of the synovial region
- Grade 3: significant vascular signals are observed in more than one-half of the intra-articular area

### Protection of the subjects’ rights

The informed consent form is formulated by the requirements of the “Declaration of Helsinki” and “Management Practices for Drug Clinical Research.” It is not only the project leader but the research team is responsible for obtaining informed consent. We will provide subjects with detailed information about the clinical study, including the research objectives, research methods, and processes, such as treatment measures, grouping, testing, the expected benefits for the subjects, possible risks, and inconveniences. At the same time, the subject’s personal information remains confidential. The subject’s participation in the clinical study is completely voluntary. At any stage of the clinical study, a participant can withdraw from the clinical research without discrimination or retaliation. Patient rights and interests are not affected. If harm or injury related to the clinical research occurs, then the subjects can be appropriately compensated. The clinical study can be conducted only after the signature of the subject or legal representative/guardian is received with the date specified. If the subject or legal representative/guardian cannot read, a witness should be present. After a detailed explanation of the informed consent form, the subject or legal representative/guardian can provide consent, and the witness can sign and date the form. Information about subjects’ participation in this study will be recorded on the study medical record/case report form. All study results that appear in the original medical records (including personal information, lab notes, etc.) will be kept completely confidential to the fullest extent permitted by law. Subjects’ name will not appear on the CRFs, only their initials and the number assigned to them at the time of their participation in the study. When necessary, drug regulatory authorities, ethics committees, or subject funding agencies are required to have access to the data of subjects participating in studies. However, they will not use the data and biological specimens of the participating subjects for other purposes or disclose them to other groups without permission.

Any modifications to the protocol which may impact on the conduct of the study and potential benefit of the patient or may affect patient safety, including changes of study objectives, study design, patient population, sample sizes, study procedures, or significant administrative aspects, will require a formal amendment to the protocol. Such amendment will be agreed upon by the project leader and research team and approved by the Ethics Committee of Shuguang Hospital Affiliated to Shanghai University of Traditional Chinese Medicine prior to implementation and notified to the Shanghai Science and Technology Commission. Finally, the modifications will be informed to subjects.

### Intervention protocols and procedures for monitoring adherence

Face-to-face adherence reminder sessions will take place at the initial product dispensing and each study visit thereafter. This session will include:

- The importance of following study guidelines for adherence to once daily study medicines
- Instructions about taking study traditional Chinese medicine granules or colchicine including dose timing, storage, importance of taking medicines, and what to do in the event of a missed dose
- Instructions about the purpose, use, care, and recycle of the drug packaging
- Notification that there will be a count of study medicines at every study visit
- Importance of calling the physician if experiencing problems possibly related to the study product such as symptoms or lost pills
- The content about any research questions of contact with the physician via Wechat or telephone

### Adverse events/serious adverse events

We will monitor the adverse events of each treatment during the trial, including acute pain and gastrointestinal discomfort. Any adverse events or reactions that are believed to be causally related to the intervention will be recorded, managed, and reported to the study coordinator. Serious adverse reactions will be reported to the ethics committee.

### Data quality control

A training will be done for all participating staff on the trial protocol, usage of the randomization, and data management systems, etc. The principal investigator will supervise the proceed of the trial at least once every month. An ethics committee will review conduct especially on the safety, rights, and well-being of the participants at the middle and the end of the trial. The auditing will be done by the Clinical Evaluation Center of the China Academy of Chinese Medical Sciences at the beginning, middle, and end of the trial.

### Data collection and management

Composition of the data and safety monitoring committee (DSMC) is composed of professional statisticians. They will perform statistical analysis, participating in the entire process from the study design and implementation to the analysis and summary. A statistical analysis plan will be developed after the completion of the study program and completion of the case reporting forms, and the statistical analysis reports will be provided after the necessary modification of the data analysis is performed as necessary during the study process. According to the project of the case report form, EpiData3.1a software will be used to establish the corresponding entry procedure and set the logical examination qualifications at the time of entry, and the database will be piloted to establish a database system dedicated to this experiment. The signed case report form and the audit statement will be given to the data administrator, who will examine the date, group criteria, culling criteria, shedding criteria, and missing values.

If there is doubt about the “data question form,” it will be returned to the monitor, and the researcher will answer and sign the question in writing and return it to the data administrator; the “data question form” should be properly stored to protect confidentiality before, during, and after the trial. The data are entered synchronously by the data administrator using a two-person entry method. The database will be checked for each item using the verification function in the EpiData3.1a software; any inconsistent result values will be reported. The original case reporting tables will be checked item by item, and 10 case reporting tables and the data in the database will be randomly selected for manual comparison to ensure that the data in the database are consistent with the results in the case reporting tables. The original CRFs and any other records will be archived for 5 years.

An interim analysis is performed on the primary endpoint when 50% of patients have been randomized and have completed follow-up. The interim analysis is performed by an independent statistician, blinded to the treatment allocation. The statistician will report to the independent DSMC. The DSMC will have unblinded access to all data and will discuss the results of the interim analysis with the steering committee in a joint meeting. The steering committee decides on the continuation of the trial and will report to the ethics committee. The trial will not be stopped in case of futility, unless the DSMC during the course of safety monitoring advises otherwise. In this case, the DSMC will discuss potential stopping for futility with the trial steering committee.

### Statistical analysis

According to the per-protocol (PP) principle, all the patients who complete and follow the study will be included in the analysis. The study will use SPSS 21.0 statistical software (SPSS Inc., Chicago, IL, USA) for statistical data analysis. If the measurement data follow a normal distribution, *t*-tests will be used for analysis, and the results will be expressed as the means ± standard deviation ($\overline{x}$± s). If the data do not follow a normal distribution, Mann–Whitney *U* tests will be used, and the results will represent the medians and quartile *M* (P25–P75) values. For the measurement of data at multiple time points (> 2) that follow a normal distribution, repeated-measurement data analysis of variance will be used. The measurement data at multiple time points (> 2) that do not follow a normal distribution will be subjected to a generalized estimation equation. Chi-square (*χ*^2^) tests will be used for the numeration data. Mann–Whitney *U* tests will be used for the grade data. For the analysis of the level data at multiple time points (> 2), a generalized estimation equation will be adopted, and *P* < 0.05 will be considered statistically significant. In the statistical analysis, the case data in which the whole treatment process could not be observed will be carried forward to the final results of the trial with the last observation data, and the intention analysis will be carried out on the efficacy and safety of the main indicators. For the analysis of missing data, we use the average filling method: if the missing value is numeric, it is filled according to the average value of other values under the same variable; if the missing value is non-numeric, take the mode of other data under the same variable to fill in. We will also perform stratified analysis to control confounding factors if necessary. Data analysis will be performed by statisticians independent of the research team.

### Ethics and dissemination

This trial protocol will be performed according to the principles laid down in the Declaration of Helsinki, has been approved by the ethics committee of Shuguang Hospital Affiliated with Shanghai University of Traditional Chinese medicine (approval no. 2018-617-46-01), and is registered at the Chinese Clinical Trial Registry (ChiCTR1900024974).

A qualified doctor will conduct the study as the principal investigator. The subject’s personal information and all the data in the trial will not be disclosed to any third party. The patients will be included in the study after receiving the study information and signing the informed consent form. The research results will be published in a peer-reviewed journal and will be disseminated in open-access, peer-reviewed journals and will be shared through oral and poster presentations at domestic conferences. All the resources will be uploaded to an online knowledge management platform. Plans for communicating important protocol modifications to researchers in time.

## Discussion

AGA is a crucial stage in the development of gout. As the deposition of monosodium urate (MSU) exceeds the normal threshold, the inflammatory reaction of the joints is rapidly increased, AGA will occur with the features of acute onset. AGA accounts for a large proportion of gout patients and causes severe physical and mental pain in patients [16]. Controlling the occurrence and development of gout inflammation is the first step in the treatment of gout.

Colchicine has been officially used as the treatment of AGA since the thirteenth century and remains one of the most recommended first-line drugs to treat AGA in many clinical guidelines. It is a classic and specific drug for AGA that can attenuate inflammation and relieve pain. Many studies [17–19] have confirmed that low-dose and high-dose colchicine can effectively reduce the pain of a gout attack, and the side effects of low-dose colchicine are better than those of high-dose colchicine. Therefore, in recent years, several worldwide gout guidelines [20, 21] have recommended low-dose colchicine or its combination with NSAIDs as a treatment for AGA. However, some patients taking small doses of colchicine have gastrointestinal reactions, such as diarrhea and nausea, and it may not control severe joint pain. Some patients limit the use of this drug because of liver and kidney damage [22, 23]. Thus, increasing the effectiveness or reducing the toxicity of colchicine, or even identifying an alternative, is difficult in the treatment of AGA.

Most traditional Chinese medicine scholars believe that gout patients mostly consume excess alcohol and greasy and surfeit flavored foods, causing dampness that blocks the middle-jiao, transform heat and accumulation, dampness, and heat accumulation in the joints, blocked blood vessels, and joint swelling and pain. Thus, moist heat arthralgia spasm syndrome is a common TCM syndrome type of AGA. AGA belongs to the category of “arthralgia syndrome” of TCM, for which the moist heat arthralgia spasm syndrome BHGZ is a commonly used prescription. Many studies have used BHGZ in the treatment of AGA, and the curative effect is significant [24–26]. However, a search of the literature from the past 20 years revealed that most of the current treatments of AGA are based on BHGZ with components added or omitted. Although these studies showed the characteristics of TCM syndrome differentiation, they did not reflect the curative effect of BHGZ, and the reference and comparability between the studies were poor. Additionally, there is a lack of high-quality RCT studies to evaluate the clinical efficacy of BHGZ on AGA. Therefore, in this study, BHGZ decoction granules will be used in the clinical treatment of AGA with a strict randomized, double-blind, placebo treatment method used to observe the clinical efficacy of the prescription for patients with the damp heat obstruction type of AGA.

Based on the current evidence, we designed this randomized, placebo-controlled, double-blind clinical trial to evaluate the efficacy and safety of AGA patients by treating them with BHGZ. This trial complies with the SPIRIT 2013 [27] and SPIRIT 2013 statements, explanations, and elaborations [28], which cover scientific, ethical, and safety issues. Regarding the AGA patients, the results of this trial may help to reduce their clinical symptoms and signs, improve their quality of life, shorten the treatment time, and quickly relieve joint inflammation. We expect that this trial will provide preliminary evidence for the efficacy of BHGZ in treating AGA patients. It will also provide substantial help for researchers, practitioners, and patients.

This study possesses some limitations. First, no international uniform standard is currently available for the musculoskeletal ultrasound evaluation of AGA in patients. Second, the study will be conducted in Shanghai, China. It remains uncertain whether the relative effects of BHGZ will be similar in other ethnic groups. Third, the specific components in BHGZ that affect the therapeutic response will require further research and exploration.

### Trial status

The protocol version number is V2.0, and the research strategy and study protocol were developed between October 2018 and June 2020. The follow-up visits and data analysis will take place from July 2020 to December 2020.

## Supplementary Information

**Additional file 1:** SPIRIT 2013 checklist. Recommended items to address in a clinical trial protocol and related documents.

**Additional file 2:** Model informed consent form

## Data Availability

Does not apply.

## Abbreviations

AGA: Acute gouty arthritis
AKP: Alkaline phosphatase
ALT: Alanine aminotransaminase
AST: Aspartate aminotransaminase
BHGZ: Baihu jia Guizhi decoction
BUN: Blood urea nitrogen
CRP: C-reactive protein
ESR: Erythrocyte sedimentation rate
Hb: Hemoglobin
IL-6: Interleukin-6
MSU: Monosodium urate
NF-κB: Nuclear factor kappa-B
NSAIDs: Non-steroid anti-inflammatory drugs
PLT: Platelets
RBC: Red blood cell count
RCT: Randomized controlled trial
Scr: Serum creatinine
TCM: Traditional Chinese medicine
TNF-α: Tumor necrosis factor-α
VAS: Visual analog scale
WBC: Blood test consists of white blood cell count
γ-GT: Gamma glutamyl
