# Supplementary material for: Effect of Baihu and Guizhi decoction in acute gouty arthritis: study protocol for a randomized controlled trial
Source: Trials. 2022 Apr 15;23:317. doi: 10.1186/s13063-022-06194-z (PMC9013133; doi:10.1186/s13063-022-06194-z)
Supplement: Supplementary file 2 — Additional file 2: Model informed consent form [file 13063_2022_6194_MOESM2_ESM.docx]

**Informed consent • Information page**

**TRANSLATED**

**Dear Sir/Madam.**

We will invite you to participate in **Effect of Baihu and Guizhi Decoction in acute gouty arthritis: A randomized, double-blind, controlled clinical study**.

Before you decide whether or not to take part in this study, please read the following as carefully as possible; it will help you to understand the study and why it is being done, the procedures and duration of the study, and the benefits, risks and discomforts you may experience as a result of taking part in this study. If you wish, you can also discuss it with your relatives and friends, or ask your doctor for an explanation to help you make your decision.

**Introduction to the study**

1. **Background and purpose of the study**

The early treatment of acute gouty arthritis (AGA) not only quickly improves the clinical symptoms but also reduces the inflammation and joint tissue damage mediated by the immune response. The aim of AGA treatment is attenuated inflammation and analgesic relief, and the main treatment drugs are Non-Steroid Anti-Inflammatory Drugs (NSAIDS), colchicine and glucocorticoids. The American College of Rheumatology (ACR) criteria and European League Against Rheumatism (EULAR) guidelines of 2016 also recommended the use of these three types of drugs. However, these drugs are associated with many adverse reactions, such as colchicine-induced diarrhea and liver and kidney toxicity; NSAID-induced gastric mucosa damage and liver and kidney injury; and glucocorticoid-induced metabolic disorders and osteoporosis. These adverse reactions greatly limit their clinical application, particularly for patients with liver and renal insufficiency and acute gout. Therefore, the exploration of additional rapidly effective and less adverse drugs in the treatment of AGA is warranted.

Chinese herbal medicine has been used in China for thousands of years. Syndrome differentiation and treatment are the main characteristics and therapeutic guidelines for traditional Chinese medicine (TCM). According to the diagnostic criteria of TCM, the analysis of clinical data is obtained from the four main diagnostic methods—observation, listening, interrogation, and pulse-taking. After years of clinical practice treating AGA, most traditional Chinese medicine scholars believe that moist heat arthralgia spasm syndrome is the common TCM syndrome of AGA. The clinical manifestations are as follows: swollen small joints of the lower limbs, painful and heated acute attacks relieved by coolness and prevention of added pressure, fever, thirst, sweating, yellow urine, red tongue with yellowish or greasy fur, and rapid pulse. The Baihu jia Guizhi decoction (BHGZ) from *the Synopsis of the Golden Chamber* can clear away heat, remove dampness, dredge collaterals and relieve pain. It is a classic prescription for the treatment for the moist heat arthralgia spasm syndrome of gout *in**ternal medicine of traditional Chinese Medicine* published by China Press of Traditional Chinese Medicine. Previous clinical studies have shown that BHGZ confers a strong benefit for treating AGA. However, the literature shows a lack of high-quality RCT research on BHGZ with respect to AGA. Therefore, in this study, we use a randomized, double-blind, controlled study with a placebo to evaluate the clinical efficacy and safety of BHGZ on the moist heat arthralgia spasm syndrome of AGA. We hypothesized that BHGZ can effectively reduce the clinical symptoms and signs, improve quality of life, shorten the treatment time, quickly relieve joint inflammation, and demonstrate a good safety profile for use with AGA patients with moist heat arthralgia spasm syndrome.

1. **Entry and exclusion criteria**

**Entry Criteria.**

1. Adult subjects with a diagnosis of AGA as stated above and with a TCM diagnosis of moist heat arthralgia spasm syndrome.
2. Males and females, aged 18-65 years, with the ability to act independently.
3. Acute onset within 48 h.
4. No other similar treatment within 14 days before the trial.
5. Signed informed consent form.

**Exclusion criteria**

1. Pregnant or lactating women.
2. Patients with stage IV–V chronic kidney disease (eGFR<30 ml/min/1.73 m^2^).
3. Allergies to the tested drugs.
4. Abnormal liver function: an ALT level that is higher than twice the normal level.
5. Severe acute/chronic organic or mental diseases.
6. Severe deformity or inability to be in the labor force because of late-stage arthritis.
7. Poor compliance and/or inability to complete the clinical observation.

**Rejection criteria**

1. Failure to meet the criteria but mistakenly admitted.
2. Failure to take medicine according to instruction during the trial, affecting the efficacy results.
3. Patients who take other traditional Chinese medicine drugs that are prohibited by the instructions of the trial, interfering with the correct evaluation of the efficacy and safety.
4. **What will be required if you participate in the study?**
5. If you meet the entry criteria and agree to participate, the following steps will be followed.
   - **Before treatment**: collect basic information (including age, gender, ethnicity, occupation; respiratory, temperature, pulse, blood pressure, tongue, history of pulse disorders, allergies, family history); Joint symptoms and signs include VAS test, joint tenderness, joint swelling and joint movement disorder; TCM evidence efficacy score; Laboratory tests include Blood test, Urine test, Stool routine, Liver function, Kidney function, Xanthine oxidase, 24-hour urine uric acid, 24-hour urine creatinine, Uric acid excretion rate, Fractional Excretion of Uric Acid (FEua), albumin/creatinine ratio, SUA, ESR, CRP, TNF-α, IL-6, IL-1, IL-10, NLRP3, NF-κB and caspase-1; ECG; Musculoskeletal ultrasonography;
   - **3 days of treatment:** perform joint symptoms and signs include VAS test, joint tenderness, joint swelling and joint movement disorder; TCM evidence efficacy score; assess drug safety.
   - **7 days of treatment:** perform joint symptoms and signs include VAS test, joint tenderness, joint swelling and joint movement disorder; TCM evidence efficacy score; assess drug safety.
   - **10 days of treatment:** perform joint symptoms and signs include VAS test, joint tenderness, joint swelling and joint movement disorder; TCM evidence efficacy score and Laboratory tests include Blood test, Urine test, Stool routine, Liver function, Kidney function, Xanthine oxidase, 24-hour urine uric acid, 24-hour urine creatinine, Uric acid excretion rate, Fractional Excretion of Uric Acid (FEua), albumin/creatinine ratio, SUA, ESR, CRP, TNF-α, IL-6, IL-1, IL-10, NLRP3, NF-κB and caspase-1; assess drug safety.
6. you will be randomly assigned to one of the two groups, you enter the drug group and the control group witha1:1probability, you and your supervising doctor do not know which group you are assigned to, in an emergency, if the understanding of the drug you are using is necessary for your treatment, your supervising doctor can always reveal the blind, so that you know the group you are assigned to and is Medications used.
7. We use biological specimens on the spot normally, and we also use an ultra-low -80ºC freezer to storage the biological specimens if it is necessary. These temperatures have been shown to maintain the viability of numerous biological assays and reagents through long-term storage. The biological specimens preserved in our study will be only used for this study, and the researchers guarantees that they will not be used for other purposes.
8. **Beneficiaries of participation in the study**

- Personal benefits: your condition may be improved by participating in this clinical study. You can get more medical advice and guidance on this disease during the trial.
- Social benefits: through a randomized, double-blind, placebo-controlled clinical study design, this study clarified the clinical efficacy of Baihu and Guizhi Decoction in the treatment of acute gouty arthritis of moist heat arthralgia spasm syndrome, which can effectively relieve the clinical symptoms of acute gouty arthritis, improve the quality of life of patients and reduce the frequency of gout attacks. It provides a new way of thinking to explore clinical treatment, excavate the new value of ancient prescriptions, give full play to the characteristics and advantages of TCM and provide evidence-based medicine basis for the formation of effective TCM treatment plan for AGA.

1. **Risks of participation in the study**

- During the experiment, there may be some discomfort during taking Baihu and Guizhi Decoction. Please tell your research doctor immediately, and he / she will deal.
- The common adverse reactions of colchicine include diarrhea, hepatotoxicity and renal toxicity. During the trial, some other discomfort may occur. Please inform your research physician immediately, and he / she will deal.
- During the study, you need to visit at the time required by the researcher, which may cause inconvenience to you.

1. **Costs, compensation and indemnification for participation in the study**

- The medications and all test used to participate in this study are free of charge.
- A one-time transportation compensation of ¥150 will be given after the completion of this study.
- If you have suffered an injury related to this study, after the expert committee has determined that you have suffered an injury, the sponsor/subject group will assume responsibility for the injury in accordance with national laws and regulations, and will provide compensation or damages for the test-related damage.
- Treatments and tests required if you also have other co-morbidities will not be covered free of charge.

1. **Is personal information confidential?**

Information about your participation in this study will be recorded on the study medical record/case report form. All study results that appear in the original medical records (including personal information, lab notes, etc.) will be kept completely confidential to the fullest extent permitted by law. Your name will not appear on the CRF form, only your initials and the number assigned to you at the time of your participation in the study. Your name will not appear on the CRF form, only your initials and the number assigned to you at the time of your participation in the study will appear, and only your initials and number will appear in relevant study summaries, articles, and public publications, if necessary.

When necessary, drug regulatory authorities, ethics committees, or subject funding agencies are required to have access to the data of subjects participating in research studies. However, they will not use the data of the participating subjects for other purposes or disclose it to other groups without permission.

1. **How do I get more information?**

- You may ask any questions about this study at any time.
- Your doctor will give you his or her phone number so that he or she can answer your questions.
- Your doctor will notify you promptly if there is any important new information during the study that may affect your willingness to continue participating in the study.

1. **Voluntary choice of participation and withdrawal from the study**

- Participation in this study is entirely voluntary on your part. You may refuse to participate in the study or withdraw from the study at any time during the study. If you choose to withdraw from the study, your benefits will not be affected and you will not be discriminated against or retaliated against as a result.
- Your doctor or researcher may discontinue your participation in this study at any time out of concern for your best interests.
- If you are withdrawn from the study for any reason, you may be counseled about your use of the study drug. You may also be asked to undergo laboratory tests and a physical examination if your doctor deems it necessary. You may also refuse, without discrimination or reprisal.
- If you choose to participate in this study, we hope that you will persist through the entire process.
- If you do not participate in this study, the study doctors will provide you with alternative treatment options, including Western medicine treatment options according to current guidelines for the diagnosis and treatment of AGA, such as NSAIDs or/and glucocorticoid.

1. **What to do now?**

- It is up to you to decide whether or not to participate in this study. You can discuss this with your family or friends before making your decision.
- Before you make a decision to participate in the study, please ask your doctor as many questions as possible until you fully understand the study.

1. **Ethics committees**

- If you have questions or need to speak to someone other than the investigator, please consult the Shanghai Shuguang Hospital Ethics Committee.
- Office of Ethics Committee: 2nd Floor, Eastern Administration, Shuguang Hospital Office of Ethics Committee
- Phone: +86-021-20256070
- Thank you for reading the above material. If you decide to participate in this study, please let your doctor know and he/she will make all arrangements for you regarding the study.
- Please keep this information.

**Informed Consent • Consent Signature Page**

Project name: **Effect of Baihu and Guizhi Decoction in acute gouty arthritis: A randomized, double-blind, controlled clinical study**

Source: Shanghai Municipal Science and Technology Commission Research Project (Subject No.: 17401971400).

**Statement of consent**

I have read the above description of this study and have had the opportunity to discuss and ask questions about the study with my doctor.

All the questions I asked were answered to my satisfaction.

I am aware of the possible risks and benefits of participating in the study. I understand that participation in the study is voluntary, I acknowledge that I have had sufficient time to consider it, and I understand that.

I can always ask my doctor for more information.

I can withdraw from this study at any time without discrimination or retaliation, and my medical treatment and rights will not be affected.

I am equally aware that if I were to drop out of the study midway through, especially if I were to drop out due to medication, it would be very beneficial to myself and the study as a whole if I informed my doctor of the change in my condition and completed the appropriate physical and physicochemical examinations.

If I need to take any other medication as a result of my illness, I will seek the advice of my doctor beforehand or tell him honestly afterwards.

I granted access to my research data to representatives of the drug regulatory authority, ethics committee or subject funding department.

I will be provided with a signed and dated copy of the informed consent form.

In the end, I decided to agree to participate in thisstudyand pledged to follow medical advice as closely as possible.

Subject's Signature: Date:

Subject contact number.

＿＿＿＿＿＿＿＿＿＿＿＿＿＿＿＿＿＿＿＿＿＿＿＿＿＿＿＿＿＿＿＿＿＿＿＿＿＿＿

I confirm that the details of this study, including their rights and the possible benefits and risks, were explained to the subjects and that they were given a copy of the signed informed consent form.

Signature of researcher: Date:

The researcher can be contacted at.
